# ‘We have no crystal ball’ - advance care planning at nursing homes from the perspective of nurses and physicians

**DOI:** 10.1080/02813432.2019.1608068

**Published:** 2019-05-24

**Authors:** Lisa Kastbom, Anna Milberg, Marit Karlsson

**Affiliations:** a Primary Health Center in Ljungsbro, and Department of Medical and Health Sciences, Linköping University, Ljungsbro, Sweden;; b Department of Medical and Health Sciences, Linköping University, Linköping, Sweden;; c Department of Advanced Home Care, Linköping University, Norrköping, Sweden;; d Department of Clinical and Experimental Medicine, Linköping University, Linköping, Sweden;; e Department of Advanced Home Care, Linköping University, Linköping, Sweden

**Keywords:** End-of-life care, advance care planning, nursing homes, goals of care, qualitative research

## Abstract

**Objective:** To investigate clinicians’ perspectives on the factors that shape the process of advance care planning in a nursing home context.

**Design:** Interviews. Latent qualitative content analysis.

**Setting:** Nine nursing homes in Sweden.

**Subjects:** 14 physicians and 11 nurses working at nursing homes.

**Main outcome measures:** Participants’ views on advance care planning (ACP) at nursing homes.

**Results:** The analysis of the interviews resulted in four manifest categories: *Exploration of preferences and views*, e.g. exploring patient wishes regarding end-of-life issues and restrictions in care at an early stage, and sensitivity to patient’s readiness to discuss end-of-life issues; *Integration of preferences and views,* e.g. integration of patient’s preferences and staff’s and family member’s views; *Decision & documentation of the ACP,* e.g. clear documentation in patient’s medical records that are up-to-date and available for staff caring for the patient, and *Implementation & re-evaluation of the ACP,* e.g. nurse following up after ACP-appointment to confirm the content of the documented ACP. The latent theme, *Establishing beneficence – defending oneself against tacit accusations of maleficence*, emerged as a deeper meaning of all the four (manifest) parts of the ACP-process

**Conclusion:** This study stresses the importance of involving patients, family members, and the team in the work with advance care planning in nursing homes. In addition, clear medical record documentation and proficiency in end-of-life communication related to advance care planning for physicians as well as nurses may also be factors that significantly shape advance care planning in a nursing home context.

**Key Points**
- Advance care planning can help patients to receive care in line with their preferences and can positively impact quality of end-of-life care.
- Our results describe a process consisting of four manifest categories and one latent theme constituting the process of advance care planning, that may be considered in education in advance care planning.
- The significance of nurses and physicians perceiving beneficence as well as fear of accusations of maleficence are important factors to contemplate.
- The study has implications for healthcare staff caring for patients near the end of their lives, in particular patients in nursing homes.

## Introduction

Advance care planning (ACP) is widely accepted as a central aspect of care for patients with severe illness [1–3], e.g. helping patients to receive care in line with their values and wishes [2]. Despite this, ACP is not practiced as widely as preferred [4]. Although patients often wish to discuss with their physician their attitudes towards, and wishes for their future care [5–7], the vast majority of patients with life-threatening diseases have never discussed such end-of-life care issues with their physician [8,9].

The reason for this underuse of ACP may have several origins relating to different parts of ACP. In the various definitions of ACP that are present in the literature [1,3], a decision-making process is most often part of the ACP definitions, and some highlight also the aspect of preparing the patient and relatives for the last days of life and the anticipated end-of-life trajectory [10,11]. Martin et al [3] identify two such goals for ACP, namely ‘to assist patients to make treatment decisions for the event of incapacity’ and ‘preparing for death and dying’ through a social process that is valuable in itself. The authors make clear that these goals overlap: establishing patients’ preferences regarding future treatment is presented as a way of ‘helping [the patient] to achieve a sense of control, relieving burdens on loved ones’ and hence is an important component of ‘preparing for death and dying’.

Martin et al’s account highlights the importance of studies examining the social process of producing an ACP, including the perspective of the clinicians. Previous research has indicated barriers to physicians initiating ACP and end-of-life discussions including their own discomfort and fear of taking hope away from the patient, feelings of losing control, prognostic uncertainty and a belief that the patient is unwilling or unable to discuss death and dying [4,12–15]. Lack of time, and aspects of organisation and leadership, also constitute barriers to ACP [15,16].

Although physicians may be restricted in initiating ACP, there are other team members around the patient [3]. Nurses often have closer and more frequent contact with the patient, and therefore may have more opportunities to initiate end-of-life discussions and identify patients’ wishes and preferences [17]. However, previous studies show that the physician in charge does not always involve nurses before making decisions involved in the ACP [17–19]. Recent research has illuminated ACP from the perspective of nurses [11,20–23]. According to Seymour et al. [21] nurses understood ACP to be an important part of good nursing care, e.g. engaging with patients to evoke care preferences, facilitate family communication and enable a shift of care focus towards palliative care.

Although there is wide acceptance of ACP as a central aspect of care for patients with severe illness (as pointed out above), its value in caring for frail older people living in nursing homes has been described as ‘a neglected research topic’ [10], despite the fact that a majority of patients living in nursing homes are old and expected to be near the end of their life. In Sweden, for example, about 45% of people who die each year die in a nursing home [24]. Furthermore, there seem to be age-related differences in terms of the quality of end-of-life care, i.e. younger cancer patients are more likely to be informed about their impending death than older patients [25], which may have impact on the process of producing ACP in nursing homes. Recent research has indicated that very few nursing home residents have an ACP [26–28].

The fact that a high proportion of deaths in Sweden take place in a nursing home makes it especially important to study ACP in a nursing home context. Therefore, the aim of this study was to investigate clinicians’ perspectives on the factors that shape the process of ACP in a nursing home context.

## Material and methods

### Settings

In Sweden, at each nursing home there is a physician in charge, often a GP who also works at a health center. When the physician in charge is on call, he or she usually has responsibility for several nursing homes in the region. Further, nurses working at nursing homes and physicians working at health centers (or hospitals) use different systems for documenting medical records, and these documentation systems are not compatible. Therefore, nurses document in one system and physicians in another, and they cannot access each other’s system.

### Participants and interviews

Inclusion criteria involved the following: being a nurse or a physician at a nursing home, being Swedish-speaking and accepting that the interview would be recorded. Participants were recruited through maximum variation sampling in terms of e.g. age, gender and time since medical/nursing degree [29]. Physicians were recruited from health centers in charge of the greatest number of nursing homes in the central area of the district chosen, and nurses were recruited from nursing homes with the most care recipients in the same district (Table 1).

**Table 1.** Background characteristics of the 25 participants.

Nurse/physician (% (*n*))	44% (11) / 56% (14)
Age mean (range)	43 years (26–64)
Age mean; nurses (range)	44 years (26–59)
Age mean; physicians (range)	45 years (35–64)
Gender men/women (% (*n*))	24% (6) / 76% (19)
Gender nurses men/women (% (*n*))	18% (2) / 82% (9)
Gender physicians men/women (% (*n*))	29% (4) / 71% (10)
Number of nursing homes participating (*n*)	9
Nursing homes, rural location (*n*)	2
Nursing homes, urban location (n)	7
Number of health centers participating (*n*)	4
Health centers, rural location (*n*)	1
Health centers, urban location (*n*)	3
Years worked since degree mean (range)	14 years (3–37)
Years worked since degree mean (range), nurses	13 years (3–36)
Years worked since degree mean (range), physicians	15 years (6–37)
Years worked at nursing home mean (range)	8 years (0.5–27)
Years worked at nursing home mean (range), nurses	5 years (0.5–13)
Years worked at nursing home mean (range), physicians	10 years (2.5-27)

In this study, we refer to a broader international definition of ACP [3], that is generally applicable to ACP, in for example nursing homes. This definition does not limit ACP to a separate encounter with the patient. An interview guide was developed by the researchers with open questions about ACP, such as ‘When should ACP be initiated?’, ‘Who should be involved in ACP?’ and ‘Who should be involved in questions regarding restrictions in care?’. Clarifying questions were asked [30]. These follow-on questions picked up on ambiguities, and were directed at understanding how these ambiguities came to make sense within the stories the participants were telling.

A physician who is also a GP (general practitioner; first author of this paper) performed the interviews. In total, 11 nurses and 14 physicians were interviewed. The interviews were conducted in 2016 and were digitally recorded and transcribed. The study was approved by the Regional Board of Ethics (Dnr 2015/385-31).

### Analysis

The interviews were analysed through latent qualitative content analysis with no predetermined categories or themes [31]. The analysis was performed using the following seven steps: (1) The transcribed interview was read through to obtain an overall impression and to get a broad understanding. (2) Segments of the texts dealing with the aim of the study were identified and meaning units were constructed. (3) The meaning units were condensed and abstracted to codes. (4) The codes were compared and sorted to categories. (5) The categories were compared to the entire interview, to make sure that the interpretation was consistent and coherent with the text as a whole. (6) The categories were compared to avoid overlapping and content descriptions were developed. (7) Quotations were used to exemplify the categories. One latent theme emerged as a deeper meaning of all the manifest categories in the analysis [31].

The preliminary categories were mainly coded by the first and second authors. The tentative categories were then discussed and revised by all the researchers. As part of the reflexivity process, the categories were validated by supplementing and contesting each other’s readings and preunderstandings [32]. To ensure the anonymity of the participants of this study, the background characteristics of the interviewee were left out when presenting quotes.

## Results

**Figure 1.**
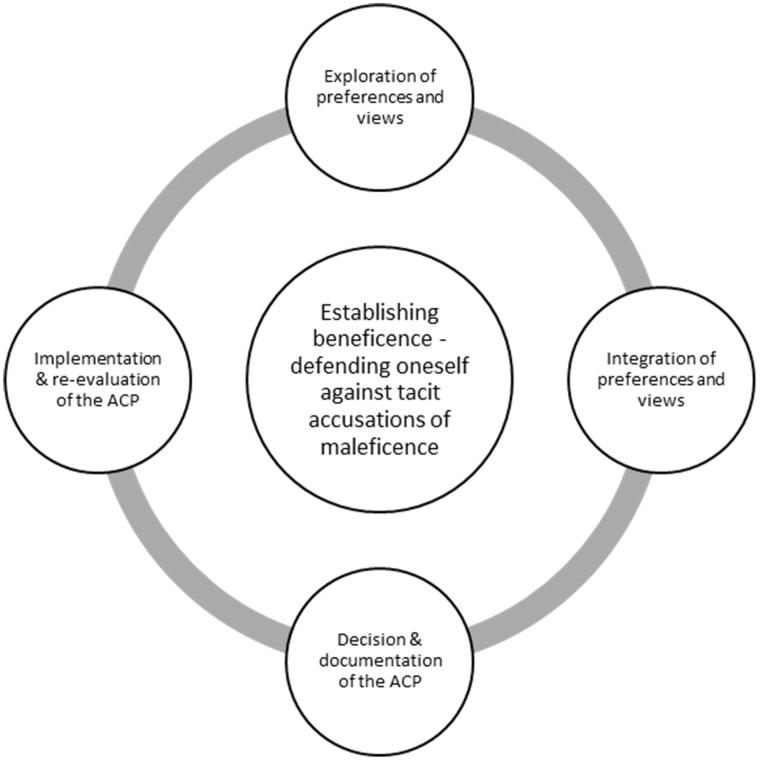
Overview of nurses´ and physicians´ perspectives on the different parts involved in the ACP-process. All the four manifest categories that emerged in the analysis related to the latent theme *Establishing beneficence – defending oneself against tacit accusations of maleficence*.

An overview of the participating nurses and physicians is presented in Table 1. When analysing the data through qualitative content analysis, a process consisting of four manifest categories and one latent theme constituting the ACP-process emerged (Figure 1). Data from both physicians and nurses were present in all the manifest categories.

### Exploration of preferences and views

One part of the ACP-process involved exploring the preferences of the patient, as well as the views of the physician, nurse and family members. According to the informants, such exploration involved questions concerning a patient’s severe deterioration or end-of-life, and what care could or could not be appropriate. To initiate such communication, exploration of the patient’s history facilitated, such as the patient’s condition, diseases, functioning and preferences as they prepared for the end of their lives.

As physicians, informants appreciated having a nurse responsible for the patient who met the patient more frequently, and therefore had a closer relationship with the patient and family members. This closer relationship meant that questions about preferences for care and end-of-life wishes could be raised with less discomfort. Physicians had confidence in the assessments made by nurses when exploring these issues with the patients.

Some of the participants highlighted difficulties in exploration of preferences, e.g. due to lack of time for explaining and coping with reactions which could emerge when they spoke to the patient and family members about sensitive issues.

For both nurses and physicians, continuity was important in enabling staff to acquire such knowledge about patients and facilitated exploration of the patient’s preferences near end-of-life. In addition, according to the participants, this was of importance not only to the nurse/physician himself/herself, but also for the patient and family members.

… Continuity is of great importance. It provides security for both the patient and relatives when they experience that someone understands the present situation before taking a stance on issues concerning restrictions in care. [Physician – interview P1]

Participants highlighted the significance of initiating ACP early enough, while the patient was still able to communicate and express his or her preferences.

Not knowing the patient could be a barrier in terms of making choices about the timing of ACP, and could sometimes lead to it being postponed. On the other hand, the participants’ experience was that, by taking time to get to know the patient, it was often possible to initiate ACP also at an early stage.

Several informants indicated the difficulties and challenges of making decisions while they were on call, that it was preferable for a physician who already knew the patient to make proactive plans for care, in order to avoid care which was not in line with a patient’s preferences.

### Integration of preferences and views

During the ACP-process, the preferences and views not only had to be explored, but also to be integrated. Nurses found it important to prepare patients as well as their family members by raising questions about wishes and preferences before an appointment with the physician, and this facilitated the process of patients and family members having spoken of these matters together – integrating their preferences and views. The role of nurses was also to make the appointment.

The physician is a bit dependent on what … how we have prepared the ground. Mostly they’re very grateful if we’ve discussed issues with relatives beforehand, so that they don’t have to go through the whole process during the limited time they’ve got. [Nurse – interview N1]

Integration of views between the nurse and the physician was also important in the ACP-process.

Discussions and support from other colleagues were appreciated by both nurses and physicians according to the informants.

### Decision & documentation of the ACP

The physicians participating in this study emphasised that the physician is the one who is responsible for the decisions about a patient’s ACP including possible restrictions in care. However, as mentioned above, the attitude of nurses towards restricted care for a specific patient is taken into consideration, as well as the attitude of patients themselves.

… The decision is always mine, but I think it’s very good to obtain the views from the patient and relatives and then of course from the nurse at the nursing home. [Physician – interview P5]

According to the informants, ACP is mainly complied with if the documentation is written clearly, with no room for misunderstanding or subjective interpretation. However, they mentioned that, if this is to happen, it is important to ensure that the documentation is available.

Informants highlighted not only to point out the restrictions in care in the ACP, but also to focus on what kind of care and efforts to do in specific acute situations, such as dyspnea and pain etc.

The informants felt that ACP documentation should be revised when the patient’s condition worsened, and some participants also expressed a desire to revise such documents more regularly, e.g. during an annual medical appointment.

### Implementation & re-evaluation of the ACP

The ACP did not only have to be implemented. There was also one part in the process that concerned re-evaluation. This part involved nurses following up with the patient and family members after appointments when ACP issues had been discussed, to address possible questions and to make sure that there was no ambiguity in terms of the limits of care and the direction it would take.

Then I can, depending on how it turns out, try to summarize, put into words//Have they understood the decision correctly? [Nurse – interview N1]

ACPs are not always complied with, according to the interviewed nurses and physicians because they are not easily accessible, or perhaps because healthcare staff are stressed and insecure in acute situations, which can result in giving the patient care against his or her previously expressed wishes.

Sometimes, when an ACP existed, it would not be used since the documentation of the ACP was not made available to the staff.

The fact that nurses in nursing homes and physicians in primary care had different medical record systems was, according to the informants, a barrier both to developing ACP and complying with it. The participants viewed the different systems for medical records as an obvious risk in terms of misjudgement or giving the patient inappropriate care.

### Establishing beneficence – defending oneself against tacit accusations of maleficence

The latent theme *Establishing beneficence – defending oneself against tacit accusations of maleficence* emerged as a deeper meaning of all the four (manifest) parts of the ACP-process (Figure 1).

All the manifest categories related to doing what was perceived as best for the patient. An important ingredient of establishing such beneficence was, according to the participants, that the patient’s preferences, in terms of what care and treatments might or might not be desired, was taken into consideration.

Each individual should have the opportunity to be in charge of his or her own life and feelings. If they feel they are very old, they are satisfied, they are happy, they are finished, they want to be left alone … Not make much effort [intensive medical care]. This is their choice. Because it’s their life… [Nurse – interview N8]

A deeper meaning of the parts of the ACP-process also involved the nurse/physician feeling uncomfortable, and having fear of causing maleficence to the patient, e.g. by speaking about death and end-of-life, by making an incorrect assessment of a patient’s condition and prognosis, in contrast to the fact that nurses and physicians are being trained to relieve and cure.

You want to relieve the suffering and you want to optimize, and you really want to cure and want the care to be good, but then … part of healthcare involves death, but when it might come and needs dealing with, then it gets uncomfortable. [Nurse – interview N3]

This theme also assumed that ACP is developed not only in the sense that the patient had the opportunity to agree to the content of the ACP, but also that family members, nurses and physicians should all be agreeing to it. Unless it was such joint, broad agreement, the interviewed nurses and physicians expressed that accusations of maleficence could arise.

You don’t want to go against the next of kin with some kind of controversy or set yourself against their wishes. In this case you want to try and explain your attitude and get them to understand it if you believe clearly that restrictions in care are needed here. Then it’s a question of establishing this somehow and … rather than disregarding the next of kin. [Physician – interview P7]

According to the participants, the nurse was seen as a defender of patient’s and family members’ preferences and views, both in discussions when forming the ACP as well as afterwards, to ensure that the patient and family members agree to the content of the ACP.

Participants mentioned that even if there was agreement on the ACP, and communication and teamwork were good, doubts could arise when in acute situations. There is always some form of uncertainty of the outcome, and doubts whether the decision, even if made in an ACP, will lead to the best consequences for the patient. Therefore, fears among the involved healthcare staff of being criticized afterwards, sometimes made them hesitant to follow the ACP. Another way to handle or defend oneself against the risk of accusations was to involve other colleagues in the decisions and to discuss difficult decisions, for example restrictions in care, with them.

… It’s difficult, but it’s not possible to avoid. We have no crystal ball. And we never have all the answers. [Physician – interview P7]

## Discussion

This study has described ACP as a process and identified four parts, namely Exploration of preferences and views, e.g. patient’s wishes regarding end-of-life issues and restrictions in care; Integration of preferences and views, e.g. integration of patient’s preferences and staff’s and family member’s views; Decision & documentation of the ACP, e.g. clear documentation in patient’s medical records; and Implementation & re-evaluation of the ACP, e.g. following up after ACP-appointment to confirm the content of the documented ACP. The latent theme, Establishing beneficence – defending oneself against tacit accusations of maleficence, emerged as a deeper meaning of the ACP-process.

Establishing beneficence was of importance in all the parts of the ACP-process. In addition, the interviews revealed strong feelings of the nurses and physicians making the wrong decisions, e.g. when to initiate ACP or determining the optimal level of care in an acute situation, and therefore perceiving fear of being accused of maleficence. Such discomfort could even hinder the initiation of an ACP-process, and this finding is supported by De Vleminck et al. in their systematic review of barriers and facilitators for general practitioners to engage in advance care planning [15].

If an increase in the proportion of nursing home residents having an ACP is wanted, it seems important that nurses as well as physicians feel support in the process of ACP including support from other staff in decisions, as well as through education. Such education could focus on identifying patients approaching last phase of life, communication with patients and families about preferences and end-of-life issues, and development of strategies to cope with the uncertainty and fear of being accused of maleficence involved in advance care planning. The relevance of education for physicians and other healthcare staff on the importance of ACP [7,33] and end-of-life care [7,33–35] has been shown earlier. It has also been reported that positive experiences with previous ACP discussions help encourage GPs to initiate ACP [12]. Prior studies on barriers for nurses in discussing ACP have shown that their lack of experience with ACP makes them feel uncomfortable in end-of-life discussions [36]. Moreover, limited education on ACP is considered a barrier to nurses discussing it [37,38], This need for education is nevertheless complicated by a lack of evidence for education and training, and there is a clear need for further studies in this area.

Furthermore, the analysis showed that the patient and family members (where appropriate), physicians, nurses and other healthcare staff were involved in different aspects of the ACP- process. In this sense, the process is based on a number of interactions between many different healthcare professions, rather than on a single appointment with the patient and family member(s). In line with this, Rapley [39] illustrates how ‘decision making is an ongoing event that often evolves over multiple encounters’. Previous studies emphasise that the physician should take the initiative and lead the ACP-process in terms of nursing homes residents [10,40]. However, other studies highlight that nurses act as facilitators in ACP, by making the voices of patients and relatives heard, for example, or by making their values known and clarifying preferences [10,22,41]. The latter view is in accordance with our results.

Different views of timing were shown to be an important aspect of ACP. Participants mentioned difficulties determining a patient’s readiness to discuss ACP while he or she was still comparatively healthy (not close to death), even if s/he was old. On the other hand, participants underlined the importance of initiating end-of-life discussions sufficiently early, while patients still had the ability to communicate their wishes and were cognitively unimpaired. The fact that nursing home residents, whose prognosis rests solely on frailty and multiple chronic conditions rather than a cancer diagnosis, could make determining the timing extra challenging.

Vleminck et al. showed that some GPs view ACP as a communication process where issues can be discussed in terms of future care options. Other GPs conceptualised ACP as a process which should be initiated late in the disease trajectory [13]. Previous studies have shown that seriously ill patients and their relatives often wait for end-of-life discussions to be initiated by their clinician [9,42], while clinicians often wait for the patient and their relatives to start this conversation [9,43]. Healthcare staff experience difficulties in determining the right time for discussing issues around end-of-life care and ACP, and this may contribute to 60–90% of patients with life-threatening illnesses never discussing end-of-life issues with their clinician [8,9,44]. Abdul-Razzak et al. [45] have shown that patients suggest an effective strategy for coping with this difficulty could involve asking them when they are ready, i.e. asking the patient’s permission. Postponing discussions on ACP until later, near the end of their life, or waiting for the patient to initiate these discussions, could result in withholding patients’ rights to be involved in their future care.

The informants experienced problems using ACPs due to the different medical record documentation systems in nursing homes and health centers (as well as in hospitals). According to the informants, these different documentation systems could hinder teamwork and jeopardise the medical safety of the patient due to lack of access of information.

## Limitations

The informants in this study were all staff working at nursing homes. An inventory of the attitudes and experiences of healthcare staff in emergency hospitals and/or nursing wards would contribute an additional perspective in terms of ACP for residents living in nursing homes and ACP for people who do not have a specific terminal diagnosis.

This study involved 25 participants: 11 nurses and 14 physicians, recruited through maximum variation sampling in terms of e.g. age, gender and time since medical/nursing degree. Generalisability is limited, as a non-probability strategic sampling method was applied [46], but the results are transferable to similar settings in nursing homes. Three different researchers were involved in the analysis of the data, thus providing an opportunity to validate the findings, which can be seen to have strengthened the results, through analyst triangulation [47]. All three authors are physicians, which could be identified as a limitation. Including a nurse, and perhaps also a non-clinician, in the research team, may have enriched the analysis process.

In conclusion, our results suggest that the different parts of the ACP-process (Figure 1), and the different identified participants should be considered in education and training in ACP. In addition, the importance of nurses and physicians perceiving beneficence as well as fear of accusations of maleficence are important factors to contemplate.

This study has implications for all healthcare staff caring for patients near the end of their life, in particular patients in nursing homes. The study has demonstrated new knowledge about ACP from the perspective of nurses and physicians, and identified possible successful structures shaping ACP in the nursing home context which in turn can lead to improvements when implemented. Further studies within this topic are needed, e.g. studies of ACP from the perspective of patients as well as family members.

### Ethics approval and consent participate

The study was approved by the Regional Board of Ethics (Dnr 2015/385-31) in Linkoping, Sweden. The consent obtained from study participants was written as well as verbal.
